# Untargeted metabolomics analysis of *Mucor racemosus* Douchi fermentation process by gas chromatography with time‐of‐flight mass spectrometry

**DOI:** 10.1002/fsn3.1042

**Published:** 2019-04-21

**Authors:** Pao Li, Hui Tang, Cong Shi, Yanhua Xie, Hongli Zhou, Bo Xia, Chunyan Zhang, Lili Chen, Liwen Jiang

**Affiliations:** ^1^ College of Food Science and Technology, Hunan Provincial Key Laboratory of Food Science and Biotechnology Hunan Agricultural University Changsha China; ^2^ Hunan Agricultural Product Processing Institute Hunan Academy of Agricultural Sciences Changsha China

**Keywords:** Douchi fermentation, GC‐TOF/MS, metabolic characterization, orthogonal partial least squares‐discriminant analysis, principal components analysis

## Abstract

Intensive study of the metabolome during the Douchi fermentation can provide new knowledge for optimizing the fermentation process. In this work, the metabolic characterization throughout the fermentation of *Mucor racemosus* Douchi was investigated using gas chromatography with time‐of‐flight mass spectrometry. A total of 511 peaks were found, and 114 metabolites were identified. The fermentation process was clearly distinguished into two main phases by principal components analysis and orthogonal partial least squares‐discriminant analysis. All the samples in the score plots were within the 95% Hotelling *T* ^2^ ellipse. Two separated clusters can be seen clearly in the score plot, which represents the two stages of fermentation: koji‐making (within 48 hr) and postfermentation (after 48 hr). Besides, clear separation and discrimination by both methods were found among different fermentation time within 15 days, while the discrimination cannot be found with more than 15 days of fermentation, indicating that the fermentation of Douchi was finished in 15 days. Due to the synergistic effect of protease and hydrolase accumulated in the early stage, proteins and other big molecular substances are rapidly hydrolyzed into a large number of small molecule components. However, the activity of enzymes decreased with the further fermentation, and some free amino acids were consumed in Maillard reaction. Therefore, there was no significant change in the content of small molecular substances after 15 days of fermentation. Furthermore, the levels of some metabolites such as alanine and lysine involved in the fermentation varied significantly throughout the processes. This study provides new insights for the metabolomics characteristics of Douchi fermentation.

## 1 INTRODUCTION

Douchi, as a large class of fermented soybean food, has played an important role in the diet of Asian countries and is becoming increasingly popular in the world. A number of functional factors in Douchi have been reported, such as effects of antioxidation, antihypertensive, and antidiabetic (Chen, Cheng, Yamaki, & Li, 2007; Chen, Wang, et al., 2011; Lu, Zhang, & Wu, 2010; Zhang, Eizo, Fan, & Li, 2010). According to different microorganisms, Chinese Douchi can be divided into three categories: bacteria type, *Mucor*‐type, and *Aspergillus*‐type. Natto is a famous bacteria‐type Douchi, and the market output value was at 3 billion US dollars every year. As a famous *Mucor*‐type Douchi, *Mucor racemosus* Douchi has become a famous health food and is gaining ever‐increasing popularity in China. However, *M. racemosus* Douchi has a longer production cycle and less production data. Much attention has been focused on the study of the Douchi fermentation (Chen, Xu, Wu, Xu, & Pan, 2011; He, Huang, Liang, Wu, & Zhou, 2016; Jiang, 2013). In the previous work, the optimal postfermentation condition for *Mucor*‐type Douchi was obtained with the help of the sensory evaluation and physicochemical indexes (Yang, Li, Chen, & Jiang, 2015). Besides, hyphenated techniques combined with electronic tongue by solid‐phase microextraction (SPME) followed by gas chromatography–mass spectrometry (GC‐MS) were used to compare the difference among three different drying methods of Douchi samples (Xie et al., 2016). In addition, the bioactive substances and overall antioxidant capacities of commercially fermented soy products were studied (Xu, Du, & Xu, 2015). The results show that phenolic profiles increased significantly after fermentation. The Douchi and fermented black bean sauce have the highest detected antioxidant profiles.

The fermentation of Douchi is very complicated. In the fermentation process, there are different kinds of microorganisms and enzymes, while the activities of each enzyme are different. The metabolites are complex, and the fermentation cycle is different. The fermentation of Douchi production was usually optimized according to the value of acidity and content of amino acid nitrogen, which are obviously not sufficient to characterize the complex process. To efficiently manipulate the fermentation process, it is necessary to give insights into the responses of the robust *Mucor* throughout the industrial process at the system level, especially at the metabolome level (Ding, Cheng, Xiao, Qiao, & Yuan, 2009). Intensive study of the metabolome during the Douchi fermentation can provide new knowledge for optimizing the fermentation process (Khakimov et al., 2017).

Changes in metabolic levels can be considered as the final response of biological systems to environmental changes (de Godoy Alves Filho et al., 2017; Ding et al., 2009; Ng'ong'ola‐Manani, Østlie, Mwangwela, & Wicklund, 2014; Shao, Zhou, & McGarvey, 2012). Metabolomics is a research field that can quantitatively measure small molecule metabolites in complex samples (He et al., 2009; Nicholson & Lindon, 2008; Spínola, Perestrelo, Câmara, & Castilho, 2015; Sun et al., 2015). For the Douchi fermentation, metabolomics focuses on comprehensive and quantitative analysis of metabolites during fermentation. A large number of high‐throughput technologies have been applied to the analysis of metabolites, such as GC‐MS (Khakimov et al., 2017; Li et al., 2019; Shao et al., 2012), liquid chromatography–mass spectrometry (LC‐MS) (Chalet, Hollebrands, Janssen, Augustijns, & Duchateau, 2018), nuclear magnetic resonance (NMR) (Marti et al., 2014; Velázquez Ríos et al., 2019; Zhu, Wang, & Chen, 2017), and capillary electrophoresis–mass spectrometry (Soga et al., 2003). Among the technologies in metabolomics, gas chromatography with time‐of‐flight mass spectrometry (GC‐TOF/MS) has been widely used due to the advantages of high resolution and sensitivity (Adebo et al., 2018; Ding et al., 2009; Salvatore, Gyong, Tobias, Cataldi, & Oliver, 2010; Sun et al., 2015; Tobias et al., 2009). With the help of the effective method, comprehensive and quantitative analysis of metabolites can be achieved, which can be used to characterize metabolic mechanism at molecular level (Zhang et al., 2017). However, the comprehensive research and optimization of the Douchi fermentation with GC‐TOF/MS technology are rarely reported so far.

The aim of the present study was to evaluate dynamics of the metabolome of the Douchi fermentation by using untargeted GC‐TOF/MS metabolomics. The metabolites of the *M. racemosus* Douchi in different fermentation time were compared with the help of principal components analysis (PCA) and orthogonal partial least squares‐discriminant analysis (OPLS‐DA).

## 2 EXPERIMENTAL

### 2.1 Materials

*Mucor racemosus* was obtained from the strain CGMCC8700 kept in China General Microbiological Culture Collection Center. Soya beans, distilled wine (alcohol content 45%), and sodium chloride were purchased from the local markets.

Douchi was prepared as previously described (Yang et al., 2015). (a) Cleaned soybean was soaked in water and then steamed for 20 min at 115°C. (b) The soybeans were cooled down to 35°C and then inoculated with *M. racemosus* rapidly, which incubated at 25°C for 48 hr. (c) The product was processed with 1% distilled wine and 8% sodium chloride, and then aged for several days (10, 15, 20, and 25 days) at 25°C. In this study, six batches of soybeans were used, while different fermentation time (0 hr, 24 hr, 48 hr, 5 days, 10 days, 15 days, 20 days, and 25 days, respectively) were investigated. So there were 48 samples in total.

### 2.2 Metabolites extraction

Sample (60 mg) was added into 0.4 ml methanol/water (3:1, v/v), and then, 20 μl of adonitol solution (1 mg/ml stock in dH_2_O) was added as internal standard. The solution was mixed in the vortex for 30 s and ultrasound treated for 5 min. Then, the solution was centrifuged for 15 min at 9810 *g*, 4°C. The supernatant (0.3 ml) was transferred into a glass vial and dried by vacuum drying. Methoxyammonium chloride solution (80 μl, 20 mg/ml in pyridine) was added into the sample and incubated for 30 min at 80°C. About 100 μl *N*,O‐bis(trimethylsilyl)trifluoroacetamide w/1% trimethylchlorosilane was added into the sample and incubated for 90 min at 70°C.

### 2.3 GC‐TOF/MS analysis

Gas chromatography with time‐of‐flight mass spectrometry system consisting of Agilent 7890 GC system with weak polar capillary column, DB‐5MS (30 m × 250 μm inner diameter, 0.25 μm film thickness), and a Pegasus 4D TOFMS (LECO Corp.) with an electron impact ionization source was employed. Only one‐dimensional GC was used in this study. In the experiment, the electron impact ionization was tuned at 70 eV and helium was used as carrier gas with an average linear velocity of 1.0 ml/min. The mass spectrometer was operated with a transfer line temperature of 270°C, ion source 220°C, and mass range from 50 to 500 amu at a rate of 20 spectra/s after a solvent delay of 370 s. The injection volume was 1 μl, and the temperature of injection was 280°C. The following oven temperature program: set the initial temperature at 50°C with 1 min, then increased to 310°C at a rate of 10°C/min, and hold 8 min (Lisec, Schauer, Kopka, Willmitzer, & Fernie, 2006).

### 2.4 Data analysis

Peak extraction, baseline correction, peak alignment, deconvolution analysis, and peak identification were achieved with the Chroma TOF4.3X software (LECO Corp.) and LECO‐Fiehn Rtx5 database. Simulation of the missing value and noise removal was achieved as previously described (Sun et al., 2015). Besides, the internal standard normalization method was used to standardize the data (Sun et al., 2015). Both of mass spectrum match and retention index match were considered in metabolites identification. LECO/Fiehn Metabolomics Library was used for the metabolite identification, and a similarity above 700 was adopted for giving the positive answer of the existence of the metabolite. Besides, the compound with the similarity <200 is defined as an “analyte,” while the compound with a similarity between 200 and 700 is considered as a putative annotation.

Principal components analysis and OPLS‐DA methods were used to display the similarity and difference with the help of the SIMCA14.1 software package (Umetrics). This software has the advantage of displaying Hotelling *T* ^2^ 95% confidence limit ellipse in the score plot to show the presence of outliers. Furthermore, the first principal component of variable importance in the projection (VIP) value above 1.0 was adopted for giving the positive answer of the existence of the changed metabolite. The remaining variables were then assessed using Student's *t* test (*t* test) method. Variable was discarded when the value of *p* was above 0.05.

## 3 RESULTS AND DISCUSSION

### 3.1 Identification of GC‐TOF/MS compounds

In this work, silylation reactions with *N*,O‐bis(trimethylsilyl)trifluoroacetamide was used. In silylation reactions, a labile hydrogen from acids, alcohols, thiols, amines, amides or enolizable ketones, and aldehydes is replaced by a trimethylsilyl group. The products are generally more volatile and thermally stable, and the major metabolites of interest may be observed by GC‐TOF/MS. The GC‐TOF/MS total ion chromatographs (TICs) with different fermentation time were obtained. In order to verify the performance of the method, the retention time and peak area of internal standard in different samples were compared. The retention times of the internal standard in different samples are stable, and the variation of peak areas of the internal standard is small (the relative standard deviation is no >5.0%). It shows that the accuracy and stability of the instrument are good. Therefore, the data of metabolites can be compared with each other in different samples. In total, 511 peaks were found and 114 metabolites were identified. The information of 511 peaks and metabolite mapping was attached as a Data S1 and S2. Besides, a clear discrimination can also be observed among the samples with different fermentation time. There were various differences in the shape and quantity of peaks among the samples with different fermentation time. Thus, these TICs identified by GC‐TOF/MS could directly reflect the differences in metabolites with different fermentation time.

### 3.2 Global analysis of metabolites with PCA and OPLS‐DA

**Figure 1:**
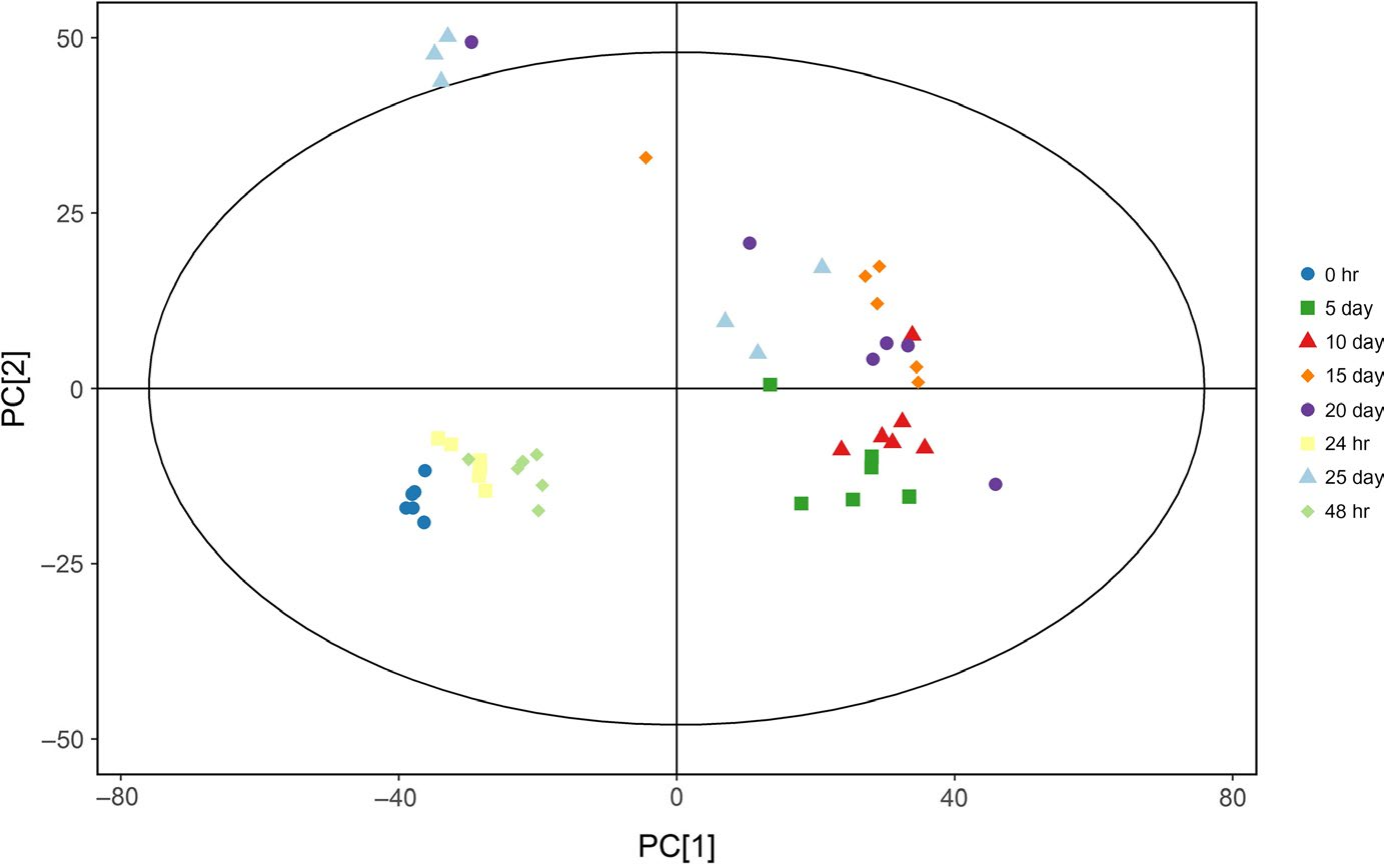
Classifications of samples with different fermentation time by principal components analysis methods

In order to discriminate the metabolites of Douchi samples with different fermentation time, PCA was performed. Figure 1 displays the classification effect of Douchi samples of different fermentation time with PCA. Ellipse represents Hotelling *T* ^2^ with 95% confidence in the score plots. All the samples in the score plots were within the 95% Hotelling *T* ^2^ ellipse. As shown in Figure 1, there was no significant difference among different batches of samples in the same fermentation time. Furthermore, the PCA analysis of GC‐TOF/MS metabolites showed significantly two separated clusters, which represent the two stages of fermentation: koji‐making (within 48 hr) and postfermentation (after 48 hr). In starter‐making stage, microorganisms can grow and multiply rapidly, produce spores, and secrete enzymes such as protease, lipase, and cellulase. In the postfermentation stage, complex biochemical reactions are carried out in anaerobic conditions with the help of the enzymes, thus forming the special flavor of *Mucor*‐type soybean sauce. After 48 hr of fermentation, a large number of metabolites were produced in the postfermentation.

**Figure 2:**
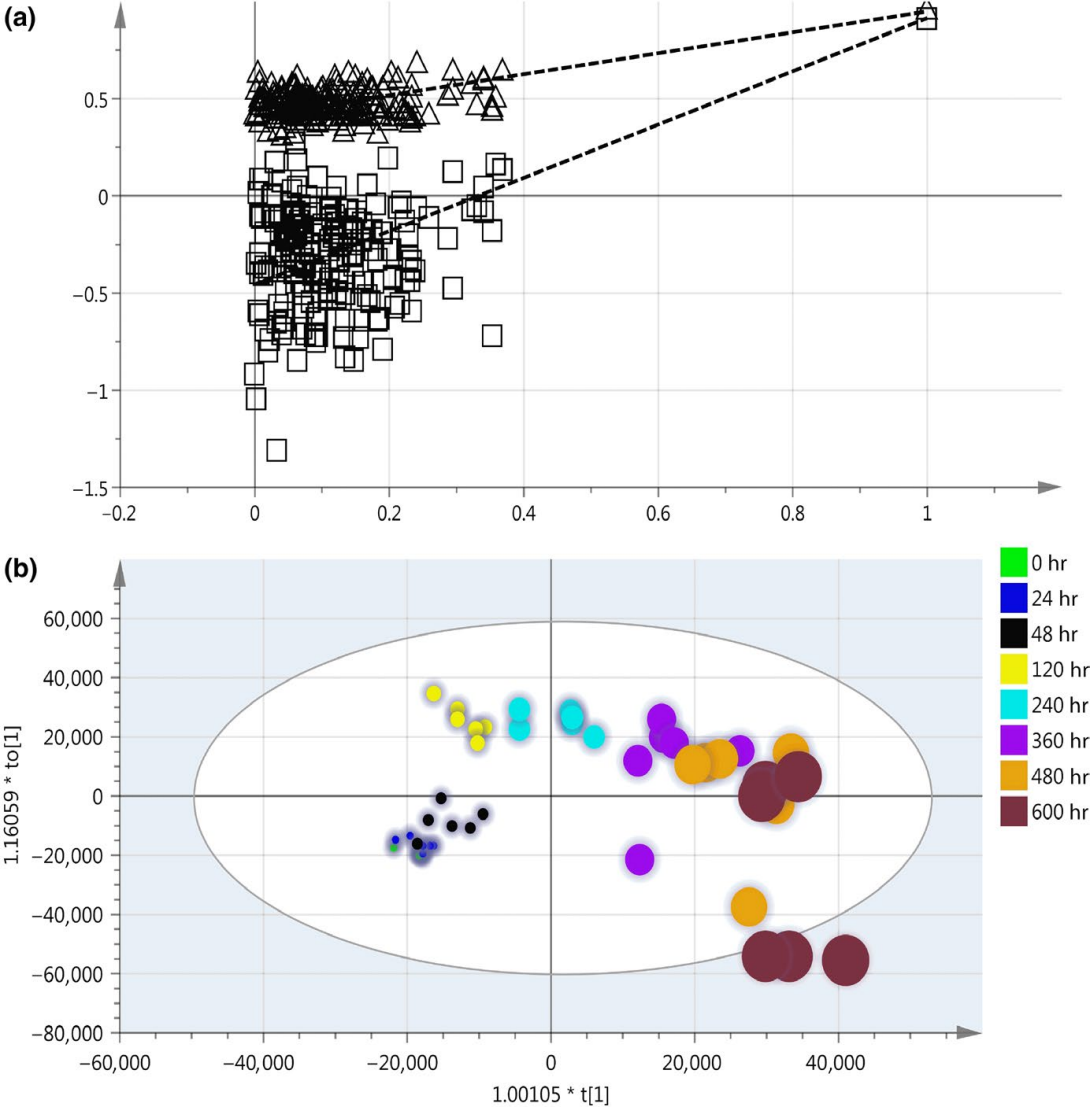
Classification of samples with different fermentation time by orthogonal partial least squares‐discriminant analysis (OPLS‐DA) method: (a) Validation plots of OPLS‐DA and (b) OPLS‐DA score plots

Orthogonal partial least squares‐discriminant analysis is a commonly used technique to improve the effect of classification. The parameter for the assessment of the OPLS‐DA model quality could be represented by the validation plots, as shown in Figure 2a. The corresponding *Q^2^Y* value of OPLS‐DA models was 0.916, and the *R^2^Y* values of the model that represent explained variance were 0.950, indicating a satisfactory effectiveness of the model. The robustness and predictive ability of the model were estimated by sevenfold cross‐validation method. In order to further validate the model, the permutation test was used. The low values of *Q^2^* intercept indicate the robustness of the models and thus show a low risk of over fitting. Figure 2b displays results by the score map of OPLS method, which represents the trend in metabolic data over fermentation time. All the samples in the score plot were inside the 95% Hotelling *T* ^2^ ellipse, whereas only two samples in 25 days of fermentation was not inside the ellipse. Separation and discrimination were clearly indicating that the OPLS‐DA model can be used to identify the difference within and after 48 hr fermentation. The levels of amino acids such as threonine, valine, glycine, phenylalanine, l‐cysteine, and alanine involved in the fermentation varied significantly. In addition, the levels of uracil and xanthine were dramatically increased. The results showed that the proteins and other big molecular substances are hydrolyzed into a large number of small molecule components with the help of enzymes in the postfermentation stage.

### 3.3 Optimization of the fermentation time

**Figure 3:**
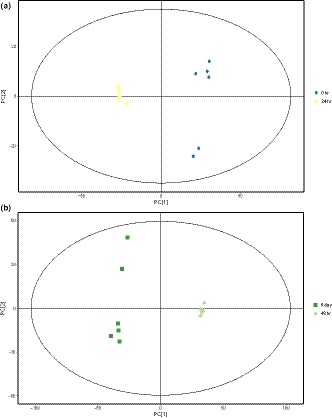
(a) Principal components analysis (PCA) score plot of the samples with 0 and 24 hr fermentation; (b) PCA score plot of the samples with 48 hr and 5 days of fermentation

**Figure 4:**
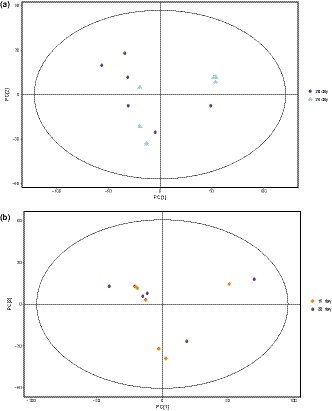
(a) Principal components analysis (PCA) score plot of the samples with 15 and 20 days of fermentation; (b) PCA score plot of the samples with 20 and 25 days of fermentation

The PCA analysis of GC‐TOF/MS metabolites showed significantly separated clusters between the samples of 0 and 24 hr, 48 hr, and 5 days of fermentation, respectively, in the PCA score plots (Figure 3a,b). However, the two groups are merged together between the samples of 15 days and 20 days, 20 days and 25 days, respectively (Figure 4a,b). The results showed that after 15 days of fermentation, there was no significant change for the metabolites in Douchi, indicating that the fermentation of Douchi was finished in 15 days. Due to the synergistic effect of protease and hydrolase accumulated in the early stage, proteins and other big molecular substances are rapidly hydrolyzed into a large number of small molecule components. However, the activity of enzymes decreased with the further fermentation, and some free amino acids were consumed in Maillard reaction. Therefore, there was no significant change in the content of small molecular substances after 15 days of fermentation.

**Figure 5:**
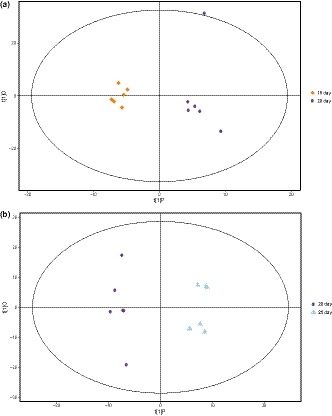
(a) Orthogonal partial least squares‐discriminant analysis (OPLS‐DA) score plot of the samples with 15 and 20 days of fermentation; (b) OPLS‐DA score plot of the samples with 20 and 25 days of fermentation

With OPLS‐DA method, similar results can also be found and significantly separated clusters between the samples of 0 and 24 hr, 48 hr and 5 days of fermentation can be found. The corresponding *Q^2^Y* values of PLS‐DA models was 0.930 and 0.941, respectively, while the *R^2^Y* values of the model that represent explained variance were 0.993 and 0.998, respectively, indicating satisfactory effectiveness of the models. However, significantly separated clusters between the samples of 15 days and 20 days, 20 days and 25 days can also be found (Figure 5a,b). The corresponding *Q^2^Y* values of PLS‐DA models was 0.099 and 0.331, respectively, indicating the classifications of OPLS‐DA method between the samples of 15 days and 20 days, 20 days and 25 days are incorrect. Therefore, it can be concluded that after 15 days of fermentation, there was no significant change for the metabolites in Douchi sample, indicating that the fermentation was finished in 15 days.

**Figure 6:**
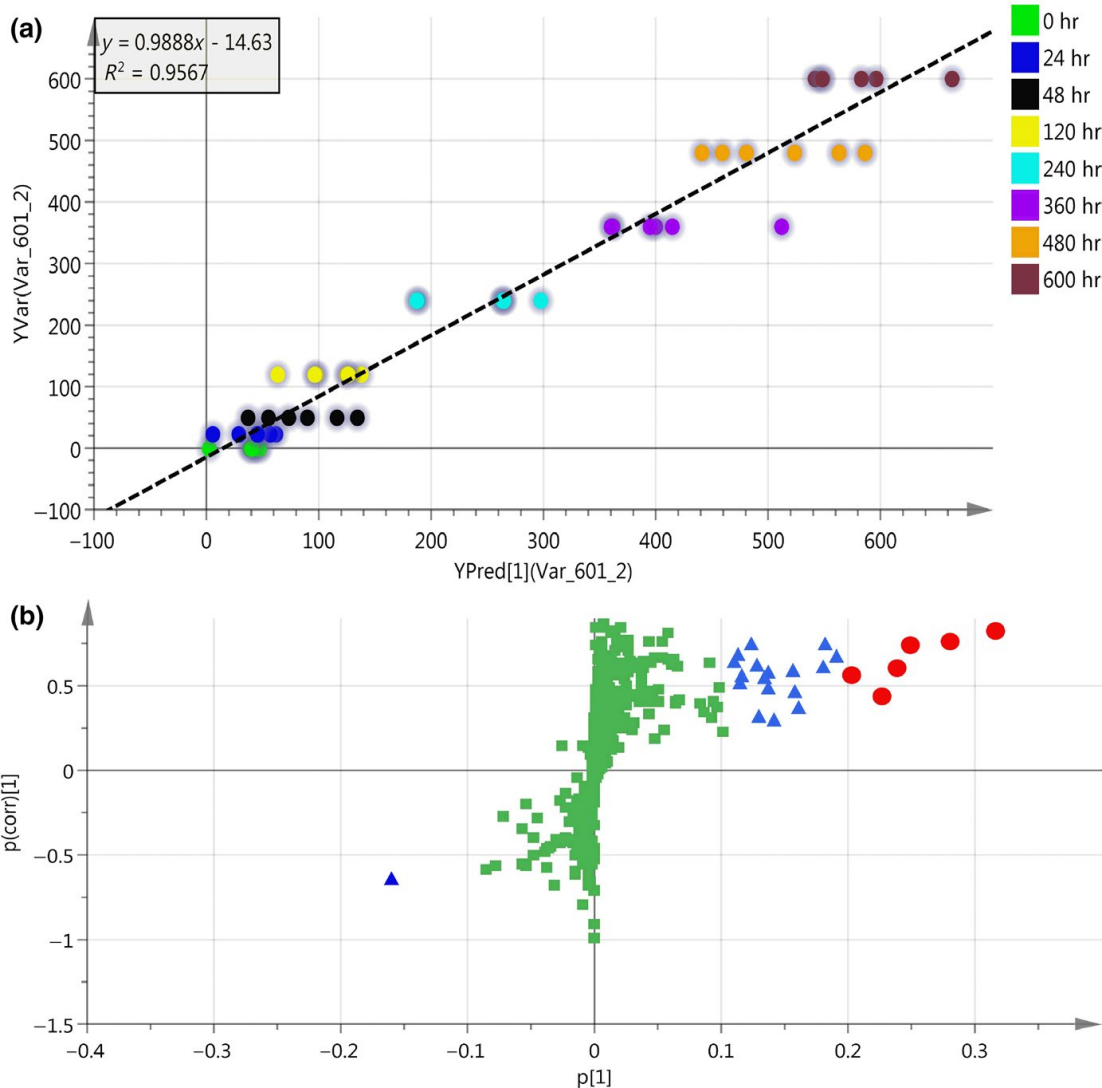
(a) Relationship of the TICs and the fermentation time with OPLS‐DA Classification of different fermentation time with OPLS‐DA; (b) S‐plot for finding the important significantly different metabolites. The blue squares represent the very significantly increased metabolites, and the red triangles represent significantly increased metabolites during the whole fermentation course. The yellow triangles represent significantly decreased metabolites during the whole fermentation course. OPLS‐DA, orthogonal partial least squares‐discriminant analysis; TICs, total ion chromatographs

Relationship between the TICs and fermentation time with OPLS‐DA can be found in Figure 6a. *R^2^* is 0.9567, while the root mean square error of prediction (*RMSEP*) is 49 hr, and the root mean square error of cross‐validation (*RMSECV*) is 70 hr. The results show that the model can extract the relationship of the TICs and the fermentation time with strong prediction ability.

### 3.4 Significantly different metabolites

For the established OPLS model, the first principal component is related to fermentation time, and thus, S‐plot was used to find the important significantly different metabolites (Lee et al., 2016). As shown in Figure 6b, blue squares represent the very significantly increased metabolites, and the red triangles represent significantly increased metabolites during the whole fermentation course. The yellow triangles represent significantly decreased metabolites during the whole fermentation course. Table 1 is the identification of significantly different metabolites. The levels of some metabolites such as alanine and lysine involved in the fermentation varied significantly throughout the processes. In addition, the levels of putrescine and myo‐inositol were dramatically increased, while the level of l‐Malic acid was decreased. In the fermentation processing of Douchi, the active phytase produced by microorganism can hydrolyze the phytic acid to the inositol and phosphate (Quan, Fan, & Ohta, 2003). Besides, the trypsin inhibitor was destroyed in the fermentation and soy protein is easier to be hydrolyzed by protease, producing a series of intermediate products, such as soy peptides and amino acids. Application of untargeted metabolomics enables unbiased analysis of metabolites which may result in discoveries that were not anticipated by production engineers and thus may lead to provide new insights into the metabolomics characteristics during the Douchi fermentation process.

**Table 1:** Identification of significantly different metabolites

Metabolites	VIPs
Significantly increased metabolites	
Myo‐inositol	7.73959
Putrescine	6.86263
Lysine	6.09263
Alanine	5.5559
Glycine	4.6789
2,6‐Diaminopimelic acid	4.43965
Valine	3.9587
Tyrosine	3.87751
Aspartic acid	3.81571
Proline	3.47342
Phenylethylamine	3.35048
Phenylalanine	3.3394
Uracil	3.29461
Isoleucine	3.17923
Ethanolamine	3.13255
Succinic acid	3.04202
Glutamic acid	2.8248
Threonine	2.35432
Isopropyl‐β‐d‐thiogalactopyranoside	1.58043
Glutaraldehyde	1.54461
O‐methylthreonine	1.50684
4‐Hydroxybutyrate	1.40658
Xanthine	1.40406
Serine	1.36458
3‐Hydroxybutyric acid	1.31852
*N*(α),*N*(α)‐dimethyl‐l‐histidine	1.22074
Malonic acid	1.11671
Maleimide	1.10606
Glycolic acid	1.08785
β‐Mannosylglycerate	1.05031
2‐Butyne‐1,4‐diol	1.04057
Significantly decreased metabolites	
l‐Malic acid	3.93109
Sucrose	2.0699
Lactose	1.89751
Ribose	1.77267
Glycerol	1.3957
Mannose	1.38651
Xylose	1.32129

Abbreviation: VIPs, variable importance in the projection.

## 4 CONCLUSION

The metabolic characterization throughout the fermentation of *M. racemosus* Douchi was investigated using GC‐TOF/MS. A total of 511 peaks were found, and 114 metabolites were identified. Clear separation and discrimination were found within 15 days of fermentation, while the discrimination cannot be found with more than 15 days of fermentation, indicating that the fermentation of Douchi was finished in 15 days. The levels of some metabolites such as alanine and lysine involved in the fermentation varied significantly throughout the processes. In addition, the levels of putrescine and myo‐inositol were dramatically increased, while the level of l‐Malic acid was decreased. Application of untargeted metabolomics enables unbiased analysis of metabolites which may result in discoveries that were not anticipated by production engineers and provides new insights into the metabolomics characteristics during the Douchi fermentation process.

## CONFLICT OF INTEREST

The authors notify that there are no conflicts of interest.

## ETHICAL APPROVAL

This study does not involve any human or animal testing.
